# Clinical Evidence against the Contagiousness of Phthisis

**Published:** 1883-07

**Authors:** E. Markham Skerritt

**Affiliations:** Fellow of University College, London; Lecturer on Medicine at the Bristol Medical School; Physician to the Bristol General Hospital


### CLINICAL EVIDENCE AGAINST THE CONTAGIOUSNESS OF PHTHISIS,

BY

E. MARKHAM SKERRITT, M.D. Lond.,
B.S., B.A., M.R.C.P.;

*Fellow of University College, London; Lecturer on Medicine at the Bristol Medical School; Physician to the Bristol General Hospital.*

---

PERHAPS no recent discovery in the domain of the science of medicine has excited so great interest as the demonstration by Koch of the existence of the *bacillus tuberculosis*, and his announcement of the results obtained in animals by inoculation of the organism. It may be assumed that most clinical observers have satisfied themselves of the association of the bacillus with phthisis; but opinion differs as to the relationship existing between this organism and the disease. Some hold that the bacillus is the exciting cause of the morbid state; and, further, accepting to the full the conclusions drawn by Koch from his experiments, are convinced that tuberculosis is contagious, and that the bacillus is the active agent in its transmission. It has been pointed out, however, that this organism may be associated with tubercular lesions without necessarily causing them—just as smoke is associated with fire, and is therefore of value in its diagnosis, though it does not cause it. Again, if further observations accord with the results of Koch's experiments (which as yet have not been fully confirmed), if it be thus established that the disease is caused by the bacillus, even then it does not follow that it is contagious. Malarial fever is known not to be capable of transfer from man to man, and yet the observations of Klebs, Crudeli, and Rozsahegyi go to prove that the disease is associated with the presence of a special bacillus, and that it can be conveyed from man to the lower animals (monkeys, rabbits and dogs) by inoculation of this bacterium.

Thus the question of the contagiousness of phthisis remains untouched by the discovery of the bacillus; and the theories which have been based upon its existence must be examined, not only from the point of view of experiments on animals, but also in the light of clinical observation. The object of this paper is to discuss from the standpoint of practical experience certain difficulties that exist in the way of the unreserved acceptance of the theory that phthisis is contagious. (It will be noted that phthisis is regarded as of tubercular origin, inasmuch as the recent observations tend to confirm the belief in the unity of phthisis with tuberculosis, which has always been held by many eminent authorities.)

The subject may be considered under the following heads:—

1.—The positive evidence in favour of the conveyance of phthisis by contagion.

2.—The negative evidence against it.

3.—Certain points of difference between phthisis and other diseases which have long been known to be contagious.

4.—Certain difficulties arising from the grouping together of affections that exhibit clinical divergencies.

5.—The bearing of the results of certain modes of treatment of phthisis upon the question under consideration.

I. *The positive evidence in favour of the conveyance of phthisis by contagion.*—It has fallen to the lot of most observers to meet with instances of the occurrence of phthisis under conditions such as to be in accordance with the theory of contagion, and especially in the case of association of individuals by marriage. It is, however, evident that phthisis is so common a disease that, on a mere calculation of probabilities, it is plain that a certain number of men who are destined to become phthisical will marry women who will also develop the disease. According to an estimate made by Dr. Beddoe, one in every seven or eight young married men has a wife who will die of phthisis. As the chances, therefore, are very much against the simultaneous onset of the disease in husband and wife, one of the two will first become phthisical, and the disease will appear in the other at some later period. And further, it is not unlikely that in such a case the concomitant circumstances of trouble and anxiety and close attendance upon the patient will be such as then to determine the onset of the malady to which the second of the pair is already doomed. It must also be remembered that the same depressing external conditions which bring on the disease in one predisposed person are very likely to do so in another, and that husband and wife are to a great extent exposed to the same injurious influences.

Leudet* gives the following statistics, respecting 56 families of the better classes, in favour of the doctrine of contagion:—Fifteen husbands were phthisical at marriage, five of the wives became affected; but of these two had relatives dying of phthisis, and one was well until *ten years* after her husband's death, so that three cases out of the

* *International Congress of Hygiene, Geneva,* 1882.

five must be excluded as doubtful. Forty-one wives were phthisical at marriage, and three of the husbands became phthisical; but here, too, one must be excluded who had lost a sister from the disease. The statistics therefore stand thus:—Two wives only out of fifteen, and two husbands out of forty-one became phthisical; a result which, according to Dr. Beddoe's estimate, it is not necessary to attribute to contagion. He further gave these details with regard to the children of the women married to phthisical husbands. Of the five wives who became phthisical after marriage, four had children, but only one lost any from tuberculosis; of the ten wives who escaped, nine had children, and five lost one or more from phthisis; so that the healthy mothers lost the most children from phthisis. Surely the fact that when both parents were phthisical fewer children suffered from the disease than when one only was affected is strong evidence against the supposition that contagion was at work; inasmuch as with a phthisical predisposition derived from both parents, and a phthisical mother to supply the specific germs, a child is evidently far more likely to fall a prey to the specific contagion than it is if the father alone is tubercular.

Poulet* records that, out of 64 cases in which one of the married couple died of phthisis at the age of fecundity, there were only two instances in which the other partner became phthisical—and in these two the disease appeared after a lapse of time and with a history of predisposition which excluded all idea of contagion.

An illustration of the fallacies to which accidental association may lead lately came under the notice of the author at the Bristol General Hospital. A man distinctly phthisical stated that he had become ill since the death of

* *Le Concours Méd.*, November, 1882.

his wife from consumption; his own family history he had no accurate knowledge of, but he was not aware that any tendency to phthisis existed. On investigation, however, it was found that the patient had enjoyed perfect health for about four years after his wife's death. If the man had chanced to become phthisical several years earlier, the case might have been quoted as another in support of the theory of contagion through marriage.

A very telling instance is narrated by Dr. W. Dale, of Lynn.* A healthy man and wife, living in a small lonely house in the country, had a family of seven apparently strong daughters and one son; two daughters were school teachers in distant places apart; two were married and also distant and living apart; and the rest lived at home. One of the teachers came home phthisical and died; after her death the youngest daughter had a severe attack of chorea, then fell a prey to acute phthisis; during her illness the other daughter engaged in teaching was brought home phthisical; a fourth daughter, living at home, was reduced by severe dyspepsia, became phthisical and died; and lastly, one of the married daughters, living under depressing conditions at a distance, developed the same disease and died. It appeared that the father had lost sisters of consumption. Dr. Dale well says that if all these cases had occurred in the same house they would have been claimed as indubitable evidence of infection.

Numerically, the cases in which contagion has been suspected of being the cause of phthisis bear a very small proportion indeed to the whole number of instances of the disease. Thus, Dr. Reginald Thompson† has met with 15 such out of 15,000; Dr. Flint with 2 cases out of

* *Lancet*, December 24th, 1881. † *Ibid*, November 6th, 1880.

690; Dr. Richardson, no cases out of 3,000. The instances brought forward by Dr. Weber in 1864 are worthy of note, especially the remarkable case of a phthisical patient who had four wives in succession die of phthisis, he himself living through it all; but it has been suggested that the disease actually at work here may have been syphilis. Cases the reverse of this, however, must be allowed full weight—as, for example, when a man has had two or even three wives successively destroyed by phthisis, he himself wholly escaping; this was long ago insisted upon by Portal.

There is one feature in some of the cases of supposed contagion between husband and wife that cannot fail to attract attention. In a certain number of the instances recorded the husband, for instance, who has developed phthisis while the wife has been dying of the disease, has made a good recovery within a comparatively short time after her death.* This is not in accordance with the progressive course of ordinary phthisis, when such a result would be very much more exceptional. Dr. Reginald Thompson† says, in speaking of such a case:—"If it had not been for previous experience of similar cases . . . I should have almost despaired of a favourable termination. . . . However, the patient improved, the physical signs cleared away, and in four months she was restored to health."

In the able paper referred to, Dr. Thompson adduces evidence in support of the view that such cases of apparent contagion are in reality of pyæmic origin, and not truly phthisical — evidence derived both from the special clinical features of the disease, which differ in

* Walsh, *Diseases of the Lungs*, 4th ed., p. 460; Reginald Thompson, loc. cit.

† Loc. cit.

many points from those of ordinary phthisis and approximate to the pyæmic type, and also from the *post mortem* appearances in one instance, where the lungs presented the characters met with in infective pneumonia,—Dr. Klein, who also examined the specimens, concurring in this opinion. Dr. Thompson says, "if further observation corroborates this evidence, it will, I think, lead to the conclusion that phthisis, in its power of communicating disease, must be looked upon, not as a zymotic disease capable of sowing itself . . . but rather as an ulcerative process capable of giving rise to pyæmia."

Many of the later features in a case of ordinary phthisis must be attributed to the septicæmic condition induced by the putrefaction which occurs in the breaking-down lung. "The thermic curves are not those of inflammatory reaction, but of putrid infection; in the pyrexial form of phthisis the evening exacerbation of temperature is due, not to a pneumonic process, but to resorption of the softened material."*

One case has been placed on record in which tuberculosis was believed to have been conveyed to a man by inoculation; but an isolated instance such as this is of no practical importance, as much wider observation would be needed before the conclusion would be warranted that the occurrence was necessarily more than a mere coincidence. Against this, also, may be set the experiments in inoculation which must have been performed inadvertently times without number during attendance upon phthisical patients and at *post mortem* examinations of those who have died of the disease, and with negative results. It is true that Laennec believed that a small nodule which appeared on his finger was a tubercular growth, the effect

* Charcot.

of inoculation; but there was no further evidence to prove that this was anything more than a simple inflammatory induration excited by the puncture. If tubercle were inoculable with such absolute certainty as Koch's experiments would indicate, a *post mortem* wound in the examination of a phthisical lung ought to be followed by the development of the disease at least as frequently as a similar inoculation with putrid matter results in blood-poisoning.

II. *Negative evidence against the contagiousness of phthisis.*—Under this heading is included the evidence that phthisis has shown no tendency to spread under certain circumstances most favourable to the development of a contagious disease.

Foremost must be placed the statistics of the Brompton Consumption Hospital. These are of so much importance that they are quoted *in extenso* as given by Dr. Pollock, Consulting Physician to the Hospital, in the Croonian Lectures for the present year on Modern Theories and Treatment of Phthisis.* The hospital has been open 36 years; it contains 240 beds. Three-fourths of the cases treated are phthisis in all stages. In the earlier years the ventilation was very faulty; the dispensary rooms were bad, and were in direct communication with the out-patient department, where from 200 to 300 consumptive patients attended daily:—

"The residents in the hospital comprise medical officer, lady superintendent, four clinical assistants (who reside for six months), sisters, nurses, and servants. All the resident medical officers are now alive, and all the matrons but one, who died in advanced age. About 150 clinical assistants have held office—eight of them are known to

* *Lancet*, April 28th, 1883.

have had consumption, generally at long periods after leaving the hospital, but none had it while resident; one had hæmoptysis before coming into residence, and in only one instance was it clearly proved that the disease was contracted in the hospital. The sisters sleep in rooms communicating with the wards and galleries, and have a system of ventilation common to the patients. The nurses sleep in rooms above the wards, but of course are all day in attendance upon the sick. In the course of 36 years only one had consumption while in the hospital. Three died of phthisis some time after leaving the hospital, two of whom were attacked many years after. Since 1867 there have been 101 nurses, of whom one died of phthisis some time after leaving. The gallery-maids scrub the wards daily. We have had 32 since 1867, but no case of phthisis occurred. Of porters, most of whom had to work in the dead-room, we have had 20, none of whom had phthisis. Of dispensers we have had 22; among them three cases of phthisis, one of whom only was ill while in the hospital; the other two contracted the disease after leaving—one from intemperate habits. There have been 29 physicians and assistant-physicians, of whom eight have died; only one died of consumption, which he had contracted before his appointment. There have been four chaplains, and nine persons in the secretary's office, but no phthisis among them."

Once a severe outbreak of erysipelas and hospital sore-throat occurred in several of Dr. Pollock's wards, when it was found that for some time there had been practically no ventilation of these wards. And yet, although they must have been living in an atmosphere teeming with the *bacillus tuberculosis*, there was an outbreak of erysipelas amongst the nurses and attendants, but no phthisis.

Compare these results with the statistics of special fever hospitals:—"Nurses in hospitals where many cases of typhus are received invariably get typhus, no matter under what sanitary conditions they are placed. There appears to be no exception to this rule. Medical men and Catholic priests in attendance upon numerous typhus cases are also almost sure, sooner or later, to get the fever."*

Next to nurses and attendants on the sick, there are none who by their occupation are more exposed to the risk of infection than are medical men. During the examination of a phthisical patient, the physician must frequently breathe his bacillus-carrying exhalations, together with chance particles of sputum expelled by cough. In spite of this, however, medical men do not suffer from phthisis in an unusual degree, and enjoy a greater immunity from the disease than do editors, dentists, architects, and public officers.†

Again, it must have fallen to the lot of all medical practitioners to witness cases in which patients dying of phthisis have been most attentively nursed by their near relatives (who presumably share any inherited predisposition that exists) under circumstances the most favourable for the occurrence of infection, with a negative result. Dr. Pollock says,‡ "I have seen many instances in which the most assiduous personal nursing of the sick, living in

* *Reynolds' System of Medicine*, Art. "Typhus Fever," by G. Buchanan, M.D.

† The following extract is from the statistics derived from records of the examination for military service in the armies of the United States during the late war of the Rebellion, of over a million men, compiled by J. H. Baxter, M.D.:—Washington, 1875.—*Rejections for phthisis per 1000 examined.*—Editors, 82.192; dentists, 65.116; architects, 63.492; public officers, 61.611; medical men, 60.729.

‡ Loc. cit.

the same room, sleeping in the same bed, and undergoing the same influences of air and lodging, of anxiety and harrass, as the sick, has failed to produce it. There have been waste of flesh and strength, loss of sleep and appetite, and all the evidences of depressed vital powers in numerous cases, but no phthisis. This, too, has occurred again and again where an inherited taint has rendered the disease most probable to invade."

III. *Certain points of difference between phthisis and other diseases which are known to be contagious.*—Of these, one of the most marked is with regard to liability to contagion, or what is commonly spoken of as *predisposition.* Those who believe in the contagiousness of phthisis, equally with those who consider it as yet "not proven," agree in this, that there is a wide divergence between phthisis and other contagious diseases with respect to its power of attack. For although, strictly speaking, a "predisposition" of body is necessary to the attack of *any* infectious disorder, yet practically it may be said that the congenial soil for the development of diseases such as typhus and typhoid fevers, scarlet fever, syphilis, and the animal poisons (as farcy and anthrax) is the *healthy system.* With phthisis, however, it is far otherwise. For if phthisis were actively contagious from man to man as are scarlet fever and syphilis, it must long ago have established its right to be classed with them amongst diseases which spread by contagion. This, indeed, appears to be a serious difficulty when the attempt is made to reconcile the results of experiments on animals with the outcome of clinical observation. For, according to these experiments, tuberculosis is inoculable, *and also communicable by inhalation of air containing the special contagium,* with the greatest certainty in the case of all animals hitherto subjected to trial—in the case of those which are not prone to spontaneous tuberculosis equally with those that have a strong tendency to its development—so that tuberculosis should be as certainly contagious as are anthrax and syphilis. Clinically, it is known that this is not the case, that air containing the products of phthisis is inhaled constantly without infection resulting, and that instances of chance inoculation must frequently arise and with negative result.

It must be allowed, then, that phthisis, if a contagious disease, differs from all others of the same class in being unable to attack those who are not peculiarly vulnerable—that, unlike the rest of the group, it requires a very specially prepared soil in which to take root, and that it will flourish in no other. And further, that this clinical fact is diametrically opposed to the results obtained by experiments on animals.

As an illustration of this point, reference may be made to cases where persons have been intimately associated—often for long periods—with phthisical patients without the development of the disease, and years afterwards have become phthisical—as in the two cases before referred to (p. 51), in one of which phthisis attacked a man four years after the death of his wife from the disease, and in the other a woman became consumptive ten years after the death of her husband from the same cause. In such cases there has been an intimate and prolonged exposure to any contagium that may exist, but with negative result; and yet, after an interval of time that precludes the possibility of infection, the same disease has developed in the survivor. If a man had been subjected in anything like a similar degree to the poison of scarlet fever, small pox, syphilis, or farcy, and notwithstanding had proved his insusceptibility to it, it is not within the bounds of probability that he would afterwards succumb to its attack.

In favour of the infectious nature of the disease it is said that, like the recognised zymotics, phthisis is much more prevalent amongst crowded communities, as in convents, barracks, and penitentiaries, than it is under opposite conditions, and that it is therefore most met with where, if contagious, it would have the best opportunity of spreading. This argument, however, tells with equal force against the theory of contagion. For nothing is more potent in bringing out existing tendencies to disease, or in developing morbid conditions due to malnutrition, than the influence of just such unhygienic conditions of life as exist in crowded communities; and it might be urged with equal force that rickets and anæmia were contagious, because they were chiefly found under similar conditions.

On closer investigation of the occupations which are more especially associated with the onset of phthisis, it will be seen that they involve the passing of a large portion of life in confined spaces, although this may not entail any actual overcrowding and therefore undue exposure to contact with phthisical persons. A reference to the statistics of the United States War Office already referred to* shows that the largest per-centage of rejections for phthisis occurred in the case of *editors*, who lead a sedentary and confined life, commonly working in rooms badly ventilated and over-heated, but who are certainly not "overcrowded" by their fellow-creatures. Far below these in the proportion of rejections for phthisis come those who work amongst somewhat similar surroundings, but *in addition* are liable to the risk of overcrowding, with the attendant greater exposure to contagion. Thus,

* See Note, p. 57.

although the rejections of editors per 1000 for phthisis were 82.192, those of bookbinders were only 24.930, of printers 21.325, and of engravers 18.405 respectively. The tables also indicate that the professions as a whole, where the element of mental strain exists to a special degree, afford a much larger per-centage of cases of phthisis than do even occupations which from their nature are commonly regarded as unhealthy, and in which the factor of overcrowding greatly increases any risk from contagion.

In a valuable paper on Phthisis in Birds, read at a recent meeting of the Bristol Medico-Chirurgical Society, Mr. G. Munro Smith states as the result of his observations on canaries that the so-called "Belgians," in which a special deformity of chest is intentionally perpetuated, when kept in confinement are almost certain to fall victims to a chronic destructive disease of the lungs; that this appears more readily if the birds are kept together (that is to say, overcrowded), but that it will arise even when complete isolation is secured; while canaries of other breeds and free from the deformity do not acquire the disease. He considers this to be the phthisis of these birds, and holds that its clinical characteristics and its *post mortem* appearances are such as to distinguish it from acute tuberculosis, to which canaries of all breeds are equally liable. These observations in comparative pathology are of much importance, as showing that, in the case of a disease which is similar to phthisis in man, and to which a strong predisposition exists, the morbid changes are developed when the element of contagion is excluded.

In a communication to the Epidemiological Society on the 4th of last April, Dr. Longstaff exhibited charts (compiled from the returns of the Registrar-General for

England and Wales) showing that the death-rate curve of phthisis—unlike those of the zymotic diseases—deviates very little from a straight line; thus resembling those of cancer, apoplexy, paralysis, convulsions and fractures. The curve of tubercular meningitis resembles it, as does (to a less degree) that of tabes mesenterica. He concludes that the mortality statistics of England and Wales afford no evidence of the contagiousness of phthisis.

Dr. Longstaff notes also the fact that during the last twenty years the death-rate from phthisis has fallen 20 *per cent.*, while there has not been a corresponding decrease in the case of those diseases which have been recognised as due to infection. This indicates that the advances made by sanitary science during that period have affected phthisis to a very special degree, and it has been proved that this diminished mortality from the disease is chiefly due to the drying of the surface of the ground and of the subsoil. In his report to the Privy Council* on the results of public works of improvement in large towns, Dr. Buchanan considers more especially the improvements which have been effected in the following respects:—In the drainage, especially of the surface; in the water supply; in the removal of excreta; in street scavenging; in the diminution of overcrowding and the enforcing of ventilation. It was found that the total mortality of towns was considerably diminished, that from the zymotic diseases included; but that the death-rate from phthisis was in many cases lowered out of all proportion. For example, at Salisbury the mortality from phthisis was as follows:—before the improvements, 44.3 per 10,000; after, 22; At Ely, before, 33 *per cent.* of all deaths were due to phthisis; after, only 16 *per cent.*

* Ninth Report to the Privy Council, by G. Buchanan, M.D.

Further, the special change to which this was referred was the *drying of the surface and the subsoil*, diminution in the death-rate from phthisis being constantly proportionate to the improvement effected in this special particular; so much so that, in several places where alterations were made in other respects but no improvement in the surface drainage could be effected, the mortality from phthisis remained as before.

It is not easy to explain these facts on the hypothesis that phthisis is infectious. There is no reason to suppose that the contagium of the disease is harboured by the soil and undergoes development in it. On the contrary, Koch's observations on the *bacillus tuberculosis* tend to show that this organism exhibits no vitality except at the temperature of the body. The only theory which can be suggested on this supposition is that exposure to damp brings the system into a state in which it proves a suitable *nidus* for the development of the contagium, so that dwellers in districts where the surface and the subsoil are insufficiently drained would fall an easy prey to the *bacillus tuberculosis*. The opponents of the theory of infection, however, consider that the interposition of the bacillus is unnecessary, and that unhealthy conditions are of themselves sufficient to excite the disease in those who are predisposed to it.

Again, a survey of the course of the disease after it has become established cannot but indicate the differences that exist between phthisis and the known zymotic diseases. Phthisis is noted for what may be called the eccentricity of its behaviour. In one case its progress will be steadily onwards to death, extending, it may be, over years, but always advancing; in another it will run its whole course within a few weeks; in another it will be checked in its career, and will altogether die out, the patient being practically cured; in yet another it will make a certain amount of progress, and then will become quiescent for a longer or shorter time, after which it will again advance; and the history of the disease may be one extending over many years, and characterised by marked irregularity of progress, by many exacerbations and remissions, but ultimately ending in death. Contrast this with the history of typhus fever or of variola; or even of glanders or farcy, which is, in its chronic form, one of the most prolonged of the contagious diseases, but which in any case runs a steady course, and ends definitely either in death or in complete and permanent recovery. The history of syphilis affords a much closer resemblance to that of phthisis in its chronicity, the uncertain duration of its lesions, and the tendency to recurrence of its manifestations; but it must be noted that this is more especially the case with reference to the tertiary stage of the disease, the phenomena of which are by some authorities considered to be sequelae of the malady rather than an integral part of it.*

IV. *Certain difficulties arising from the grouping together of affections that exhibit clinical divergencies.*—Space does not permit of a detailed consideration of the various diffi-

* It is hardly necessary to point out, in reply to an argument that is sometimes used, that similarity in the minute structure of neoplasms does not necessarily imply similarity in the exciting causes—that tuberculosis is not necessarily contagious because its new growths, like those of farcy and glanders, belong to the group of granulomata, any more than simple inflammation is contagious because it leads to the formation of the granulation tissue which is the type of the granulomata. Nor is it any more logical to draw an analogy between acute tuberculosis and typhoid fever because clinically cases occur in which it is almost impossible to distinguish between them, than it is to group together acute tuberculosis and the other and varied morbid conditions which it may simulate in turn.

culties attendant upon the unreserved acceptance of the conclusions based upon Koch's experiments, by which is necessitated the grouping together of conditions differing widely in their clinical history. Acute miliary tuberculosis and strumous enlargement of lymphatic glands, chronic pulmonary phthisis, strumous synovial degeneration of joints, and the pearl-disease of cattle, are all, according to Koch, convertible terms of the same disease, are all due to one and the same contagium.

It would be possible to indicate many points—such as the morphological distinctions between human tuberculosis and pearl-disease, as insisted upon by Dr. Creighton,* and, later, by Schottelius; the sequence of acute tuberculosis upon old-standing strumous joint disease or caries of the spine in one case—the strict and permanent localisation of the disease in an affected joint in another, although the extensive secondary lardaceous degeneration of internal organs shows the profound influence of the malady upon the system—the extreme rarity of the occurrence of strumous joint disease as a complication of phthisis; it were easy to multiply such illustrations of the practical difficulties to which the theory of the unity of these various conditions gives rise.

V. *The bearing upon the question under consideration of the results of certain methods of treatment.*—Brief reference may here be made to an argument that has been derived from the supposed effects of two modes of treatment directed against the bacillus of tubercle—the treatment by antiseptic inhalations, and the treatment by residence at high altitudes.

First, with regard to the inhalation of antiseptics, such as creasote and carbolic acid. In spite of the assertions

* Bovine Tuberculosis in Man, by C. Creighton, M.D. 1881.

of some observers, it has not been established that this method of treatment has any power in controlling the progress of phthisis. And indeed it is not likely that any material effect can be produced upon organisms which exist in the air-spaces (let alone those in the tissue of the lung itself), when the very slight degree of volatility of the substances employed is borne in mind.*

Again, with reference to the influence on phthisis of residence at high altitudes, there is much discrepancy of opinion. It must be remembered that the rare occurrence of the disease amongst the inhabitants of such regions has no immediate bearing upon the question, inasmuch as in the case of established phthisis the specific germs are supposed to be present in the lungs already; and the theory that the pure air is fatal to the bacilli that exist in the tissues is not sound, as viewed in the light of "Listerism." For it is well known that, when putrid decomposition has once set in, a wound cannot be made aseptic even though kept in an atmosphere of carbolic acid, and that the thorough application of a strong solution can alone be relied upon to secure the desired result. Pure air alone is not an efficient germicide; the germs already in possession must be destroyed, and then, if none exist in the locality, no more will be introduced.

* Dr. Hill Hassall has shown [*Lancet*, May 5th, 1883] that carbolic acid and creasote, the substances most commonly used for inhalation, are but very slightly volatile at ordinary temperatures; and that even iodine, which is a much more active disinfectant, becomes converted into an inert iodide before it can reach the ultimate lung-structure. Further, Dr. Baxter has proved [*Report on Disinfectants, Public Health Reports of the Local Government Board, 1875*] that 2 °/₀ of pure carbolic acid must be added to vaccine in order to destroy its power, and that the same amount is also necessary in the case of the virus of glanders; so that, supposing the tubercular poison to have the same power of resistance, in order to destroy the bacilli in any portion of sputum it would be necessary to add to it an equal quantity of a 1-in-25 solution of carbolic acid.

The following fact, however, is often overlooked:—that, although phthisis is rare at high altitudes, yet "*tuberculous deposition is by no means very uncommon in certain other organs of natives of the highest habitable regions.*"* This tends to show that the rarity of pulmonary phthisis amongst the inhabitants of such altitudes is due to the massy normal development of the lungs, which endows these organs, the favourite seat of the disease, with a greater power of resistance, rather than to the absence of tuberculosis from those regions. This is in accordance with the fact that musicians who play wind instruments, and in whom the capacity of the chest becomes unusually great, are peculiarly exempt from phthisis. In the statistics of the United States armies before referred to the rejections of musicians per 1000 for this disease were only 12.049, as against the 82.192 of editors.

It has been said that favourite health resorts become "contaminated" by the crowding of cases of phthisis; but there is not sufficient evidence to show that their apparent deterioration is due to any other cause than non-specific overcrowding with its usual unsanitary concomitants; and it must also be borne in mind that when a health resort becomes "fashionable" patients in all stages of disease will flock to it without reference to its special fitness for their individual condition, and that the reputation of the locality suffers in consequence.

A detailed consideration of the results which have been obtained by experiments on animals, and a discussion of the conclusions which have been drawn from them as to the exact relationship of the bacillus to tuberculosis, are not within the scope of this paper. It may, however,

* Walsh, loc. cit., p. 465.

be pointed out that it is not safe to reason directly from the lower animals to man, or even from animals to animals. For a fungus which kills one species of mouse is entirely inactive in another;* mice are highly susceptible to anthrax, while rats enjoy almost complete immunity from it; the herbivora as a whole are very prone to anthrax, whereas in the carnivora it cannot be induced, even by inoculation, with any certainty; the so-called rabbit-septicæmia is very fatal to rabbits and mice, while guinea-pigs and rats are not in any way affected by it; inoculations from relapsing fever have been successful only in monkeys; and even human saliva, injected into rabbits, causes a form of septicæmia.†

Further, there are important discrepancies between the results obtained by different observers. For example, in his celebrated observations on the induction of tuberculosis in dogs by the inhalation of phthisical sputa, Tappeiner made his "control" animals, for the sake of comparison, inhale the material from *strumous glands*, and in every case without the production of tuberculosis. And Carl Salomonsen,‡ in a series of inoculations with "non-tubercular matter giving negative results," includes two experiments with matter from a *caseous gland from a scrofulous child*. In all these cases, however, tuberculosis should have been produced, inasmuch as the material used for inhalation in the one series and for inoculation in the other contained, according to Koch, the specific *bacillus tuberculosis*. Vargunin, indeed, has made dogs inhale atomised phthisical sputa, emphysematous sputa, and suspended mechanical matter, with similar results in all;

* Koch. † Raynaud, Pasteur.

‡ Quoted by Watson Cheyne in his Report to the Association for the Advancement of Medicine by Research, on the Relation of Micro-organisms to Phthisis, *Practitioner*, April, 1883.

that is, the induction of simple disseminated broncho-pneumonia, without any indication of the existence of an infectious disease.

Again, evidence varies widely as to the constancy of the association of the bacillus with phthisical lesions and its relation to the progress of the disease. According to some observers, this organism is always present in tubercular growths, and its numbers are proportionate to the severity of the condition existing; according to others, the bacillus has not been found in a certain number of instances,* and in any case it is by no means necessarily present in proportion to the amount of tubercular disease. The general body of evidence goes to show that the organism is chiefly met with in caseous matter—that is, where the products of tuberculosis have undergone degeneration; and it has not yet been proved that it is the cause of the disease, rather than a concomitant which finds in tubercular tissue a congenial soil for its growth.†

The diagnostic value of the bacillus depends upon its distinctive reactions to colouring-matters, as insisted upon by Koch. This observer himself, however, allows that the bacteria of leprosy take the same stain; Cramer and Lichtheim have found bacilli in healthy stools with the same colour-reaction. Finkler and Eichler state that other bacilli, not tubercular, behave in the same way; and that even nuclei, if not kept too long in the acid, may regain the blue colour after washing in distilled water. Lastly, Spina, Stricker's assistant at Vienna, states that

* The assumption made by Watson Cheyne (loc. cit.) that only those structures are true tubercle in which the bacillus exists appears to be a begging of the question.

† The supposed *micrococcus gonorrhϾ* has been shown to be nothing but the *micrococcus ureæ*.

*putrefactive bacteria* stain with Koch's reagents; and this fact is vouched for by no less an authority than Stricker himself. This, if true, "destroys the main argument advanced by Koch in favour of the specific character of the tubercle-bacillus."

*Conclusions.*

1. That evidence derived from experiments upon the lower animals must be received with caution; and that it does not follow that a disease which is contagious in these animals is contagious also in man.

2. That it has not been conclusively proved that the bacillus is the cause of tuberculosis rather than associated with it as a secondary phenomenon. And further, that if the disease be shown to be due to the bacillus, even then it is not necessarily contagious; as malarial fever is not contagious, although it has been induced by inoculation of an associated organism.

3. That clinical experience is strongly opposed to the theory that phthisis is a contagious disease in the ordinary sense of the term.

4. That there is not sufficient evidence of the actual occurrence of phthisis in man by contagion.

The contagiousness of phthisis is as yet "not proven;" and it behoves the scientific observer not only to listen to the arguments which are adduced in support of an attractive theory, but also to give due consideration to the weighty evidence to which this theory is opposed.

